# Supplementary material for: A Critical View of the Application of the APEX Software (Aqueous Photochemistry of Environmentally-Occurring Xenobiotics) to Predict Photoreaction Kinetics in Surface Freshwaters
Source: Molecules. 2019 Dec 18;25(1):9. doi: 10.3390/molecules25010009 (PMC7017383; doi:10.3390/molecules25010009)
Supplement: Supplementary file 1 [file molecules-25-00009-s001.zip › 3.2.4_gcc-4.4.0.zip!/3.2.4_gcc-4.4.0/3.2.4_gcc-4.4.0/doc/octave/html/interpreter/Block-Comments.html]

Block Comments - Untitled


Next: Comments and the Help System,
Previous: Single Line Comments,
Up: Comments


---

#### 2.7.2 Block Comments

Entire blocks of code can be commented by enclosing the code between
matching #{ and #} or %{ and %} markers.
For example,

```
     function quick_countdown
       # Count down for main rocket engines
       disp(3);
      #{
       disp(2);
       disp(1);
      #}
       disp("Blast Off!");  # Rocket leaves pad
     endfunction
```

will produce a very quick countdown from '3' to 'Blast Off' as the
lines "`disp(2);`" and "`disp(1);`" won't be executed.
